# The effect of local application of nonivamide/nicoboxil (Finalgon^Ⓡ^) on random-pattern skin flap survival after nipple-sparing mastectomy and breast reconstruction with tissue expander

**DOI:** 10.1016/j.jpra.2025.11.024

**Published:** 2025-11-22

**Authors:** Anja Imsirovic, Anton Schwabegger, Agnese Nitto, Marie Emilia Casari, Dolores Wolfram-Raunicher, Monika Lanthaler

**Affiliations:** Department of Plastic, Reconstructive and Aesthetic Surgery, Medical University of Innsbruck, Innsbruck 6020, Austria

**Keywords:** Finalgon, Breast surgery, Nipple sparing mastectomy, Skin flap perfusion, Reconstructive surgery, Wound healing

Over the past 3 decades, reconstructive breast surgery—particularly following mastectomy—has increased dramatically. Immediate breast reconstruction rates have risen from about 10 % in the 1980s to nearly 90 % today. Among the various surgical techniques, nipple-sparing mastectomy (NSME) has become widely practiced when oncologically feasible. Despite these advances, one of the major challenges remains poor skin flap perfusion after mastectomy, which can compromise wound healing and increase the risk of implant or expander loss.

Traditionally, intraoperative evaluation of flap viability relies primarily on the surgeon’s clinical assessment. However, nowadays the value of indocyanine green (ICG) angiography, a technique that provides a reliable real-time assessment of perfusion and can help reduce the incidence of skin necrosis, is being widely discussed. When poor perfusion is detected, the first corrective step is usually partial deflation of the tissue expander to relieve pressure. In more severe cases, necrotic tissue must be excised, and the expander may need to be replaced.

Preventing necrosis in random-pattern flaps involves multiple physiological strategies—enhancing capillary formation, improving microcirculation, reducing oxidative stress, and minimizing reperfusion injury. These mechanisms can potentially be supported pharmacologically by topical agents that enhance local blood flow.

One such agent is Finalgon^Ⓡ^ (Bender and Co., Vienna, Austria), a topical rubefacient cream containing nonivamide (a capsaicinoid) and nicoboxil (a nicotinic acid ester). Traditionally used for musculoskeletal pain relief, arthritis, and rheumatism, it induces vasodilation and warmth through its combined pharmacologic actions. Recent studies^1^, ^2^, ^3^, ^4^, ^5^ have further clarified its mechanisms and confirmed its safety profile.

Nonivamide promotes vasodilation by stimulating the release of substance P and calcitonin gene-related peptide (CGRP)—two neuropeptides that increase vascular permeability and local blood flow. Substance P, produced by macrophages and lymphocytes, also plays a regulatory role in immune responses. Nicoboxil, the nicotinic acid ester in Finalgon^Ⓡ^, induces skin flushing by promoting prostaglandin D2 (PGD2) release, which further mediates vasodilation and immune cell activation. Together, these compounds create localized hyperemia and warmth that can enhance perfusion in ischemic tissues.

Clinical trials^1^, ^2^, ^3^, ^4^, ^5^ have shown that Finalgon^Ⓡ^ is generally well tolerated, with transient erythema, warmth, or mild burning as the most common side effects. Importantly, there are no significant systemic adverse effects, making it a safe option for topical use. Prior experimental research into topical anti-ischemic agents—such as nifedipine, nitroglycerine, and trolamine salicylate—has largely focused on animal models, with limited translation to clinical practice. Thus, evaluating the potential of Finalgon^Ⓡ^ in human surgical cases offers an innovative direction.

Our aim was to explore the off-label use of Finalgon^Ⓡ^ in managing impaired skin perfusion following NSME with immediate breast reconstruction. The rationale was derived from the product’s established vasodilatory effects on muscle and skin tissue, suggesting potential benefits in improving flap viability.

In our standard postoperative care protocol, minor perfusion impairment was typically managed with heparinoid cream (Hirudoid^Ⓡ^), which can improve blood flow modestly. However, in cases of more severe perfusion deficits, the improvement with Hirudoid^Ⓡ^ was limited. Therefore, we started with topical application of Finalgon^Ⓡ^ as an adjunct therapy. Application of the cream was initiated when impaired skin perfusion was observed. If necessary, the expander was partially deflated to relieve pressure on the compromised area. All dressing changes and postoperative assessments were performed by the same surgeon to ensure consistency.

Daily clinical observation and documentation (COD) were carried out to monitor perfusion progress and determine if surgical revision was required. Patients were discharged 5–7 days postoperatively, with continued outpatient follow-up for at least 3 weeks. Skin perfusion gradually improved over this period, and routine expander inflation was resumed 3–4 weeks after wound healing was complete.

Between December 2023 and November 2025, we successfully treated 12 patients with Finalgon^Ⓡ^, avoiding the need for further revision surgery. A further four patients were also treated but ultimately required operative wound necrosis excision. However, our clinical observations showed that the extent of the necrosis that had to be resected was smaller. After 1 year, all reconstructions remained stable, and patients successfully completed the second stage of their reconstruction without complications. These findings suggest that Finalgon^Ⓡ^ could serve as a valuable adjunct therapy for patients with moderate perfusion impairment—cases that traditionally fall between observation and surgical revision.

While these results are encouraging, we emphasize that the data are preliminary and drawn from a limited experience, thus showing promising results. Further controlled studies using imaging techniques such as ICG angiography or Doppler flow measurement are necessary to objectively quantify the perfusion improvements observed.

In conclusion, Finalgon^Ⓡ^ may significantly improve skin flap perfusion following NSME, thereby reducing the need for surgical revision and enhancing overall reconstructive outcomes. While larger studies are required to confirm these results, preliminary data suggest that Finalgon^Ⓡ^ could be an effective and low-risk addition to postoperative management protocols for breast reconstruction patients with impaired skin perfusion (Figure 1, Figure 2).

**Figure 1:**
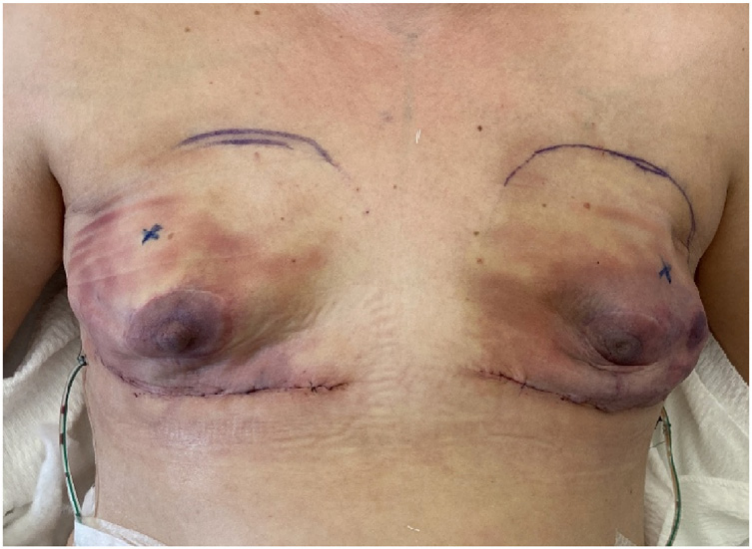
Patient on first postoperative day after NSME.

**Figure 2:**
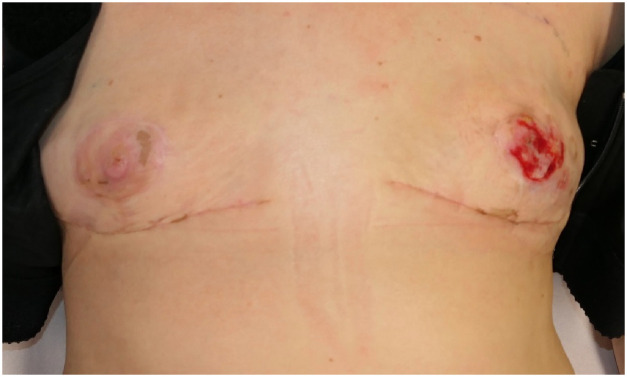
Patient on 25th postoperative day, 3 weeks after discharge.

## Author contributions

All authors have made substantial contributions to: Conception and design of the study, Data acquisition, Data analysis and interpretation, Drafting of the article, Critical revision of the article for important intellectual content, Final approval of the version to be submitted.

## Funding

None.

## Declaration of competing interest

None declared.

## References

[1] Moro C., Bass J., Scott A.M., Canetti E.F.D. (2017). Enhancing capillary blood collection: the influence of nicotinic acid and nonivamide. J Clin Lab Anal.

[2] Papaliodis D., Boucher W., Kempuraj D. (2008). Niacin-induced "flush" involves release of prostaglandin D2 from mast cells and serotonin from platelets: evidence from human cells in vitro and an animal model. J Pharmacol Exp Ther.

[3] Davis R.E., Wachholz J.H., Jassir D., Perlyn C.A., Agrama M.H. (1999). Comparison of topical anti-ischemic agents in the salvage of failing random-pattern skin flaps in rats. Arch Facial Plast Surg.

[4] Huemer G.M., Wechselberger G., Otto-Schoeller A., Gurunluoglu R., Piza-Katzer H., Schoeller T. (2003). Improved dorsal random-pattern skin flap survival in rats with a topically applied combination of nonivamide and nicoboxil. Plast Reconstr Surg.

[5] Huemer G.M., Froschauer S.M., Pachinger T., Kwasny O., Schoffl H. (2009). A comparison of pretreatment with a topical combination of nonivamide and nicoboxil and surgical delay in a random pattern skin flap model. J Plast Reconstr Aesthet Surg.

